# Online disinhibition mediates the relationship between emotion regulation difficulties and uncivil communication

**DOI:** 10.1038/s41598-024-81086-7

**Published:** 2024-12-03

**Authors:** Aleksi H. Syrjämäki, Mirja Ilves, Thomas Olsson, Joel Kiskola, Poika Isokoski, Anna Rantasila, Gary Bente, Veikko Surakka

**Affiliations:** 1 Research Group for Emotions, Sociality, and Computing, Faculty of Information Technology and Communication Sciences, Tampere University, Tampere, Finland; 2 Technology and Social Interaction Research Group, Faculty of Information Technology and Communication Sciences, Tampere University, Tampere, Finland; 3 Lappeenranta-Lahti University of Technology, Lappeenranta, Finland; 4 Department of Communication, Michigan State University, East Lansing, MI USA; 5 Tampere University, Tampere, Finland

**Keywords:** Uncivil communication, Online disinhibition, Computer-mediated communication, Social media, Online discussion, Digital emotion regulation, Human behaviour, Information technology

## Abstract

Uncivil online communication is a widely problematized cultural by-product of computer-mediated communication that, however, remains theoretically underexplained. While previous research shows that personal tendency for uncivil communication is partially influenced by individuals’ personality and empathy skills, the factors of inter-individual variation remain largely unknown. The present study examined individuals’ emotion regulation skills as a possible predictor of uncivil communication. Online survey respondents (*N* = 215) reported if they had engaged in uncivil communication and filled in scales measuring emotion regulation difficulties, use of different emotion regulation strategies, and various individual traits. The results show that emotion regulation difficulties were associated with high levels of online disinhibition. This, in turn, was associated with reports of uncivil communication. The mediation effect was observed even when controlling for personality and empathy. The results suggest that individuals’ emotion regulation difficulties may be an underlying psychological factor behind harmful online communication. These findings call for research and development of means to support emotion regulation in online interactions.

## Introduction and theoretical framework

While the internet has revolutionized interpersonal communication, the computer-mediated and often text-based form of communication has introduced new social and cultural problems. One persistent issue on many online platforms is the prevalence of expressions of hatred, personal attacks, and other forms of uncivil communication^1^. Uncivil communication can have various detrimental consequences, such as hindering constructive discourse^2^, polarizing opinions in the society^3^and lowering the perceived quality of online platforms^4^. Previous research has shown that specific individual traits such as narcissism and low empathy are associated with online incivility^5–7^. However, we still lack comprehensive understanding of the individual-level factors that predict engagement in such behavior at large.

We suggest that a key gap in the literature relates to individual differences in emotion regulation processes. Experimental studies have demonstrated that users’ negative emotions are one of the causal mechanisms that trigger uncivil online behavior^8,9^. Following this, it seems likely that individuals’ online behavior could be influenced by how they regulate their emotions but to the best of our knowledge the associations between individuals’ emotion regulation skills and uncivil communication have not been studied. Emotion regulation seems to be more difficult in textual computer-mediated communication as compared to face-to-face situations. Research on media studies implies that the attenuation of emotion regulation online is associated with factors identified in the online disinhibition effect^10^. For example, anonymity, invisibility, asynchronicity, and minimization of authority in online communication may be associated with the phenomenon. Emotion regulation is theoretically linked to online disinhibition because poor regulation can diminish self-control in digital spaces, where the typical social norms regulating the behavior are weakened.

In this study, we conducted an online survey to examine whether individual differences in emotion regulation (i.e., perceived difficulties in emotion regulation and use of different emotion regulation strategies) are associated with uncivil online communication. It is noted that we mostly focused on incivility forms related to speech-based norms, and only indirectly addressed violations of the inclusion-based norms^11^. The findings provide new information about the associations between uncivil online behavior and emotion regulation and have practical significance in the development of interventions that could support emotion regulation in online communication.

We will next present the theoretical and empirical background of the current study, describing how the concepts measured in the study are related to uncivil online behavior according to prior literature.

### Theoretical background

#### Uncivil Communication

Uncivil expressions, toxic language, and antisocial behavior^1,12^ are common on social media platforms. Coe et al.^1^reported that approximately one-fifth of examined online news comments contained incivility. Surveying social media interactions of UK parliament members, Akhtar and Morrison^13^ found that every respondent reported having been targets of online trolling as one form of antisocial behavior. These findings suggest that many behaviors that would be considered inappropriate in face-to-face interactions are common in online contexts.

While it is accepted that online incivility is common and has wide societal repercussions, the concept of incivility is difficult to define^1,6^. The concept can be divided into *personal-level incivility*, which refers to impoliteness and rude choices of words, and *public-level incivility*, which refers to a lack of deliberativeness and reciprocity, hence threatening democracy^11,14^. The former term can also be referred to as *uncivil discourse* and the later as *intolerant discourse*which may not always be perceived as uncivil, but individuals or groups for example minorities, are attacked in ways that is morally questionable and threatens democracy^15^. In this study, the use of the concept of incivility focuses more on acts that violate interpersonal politeness norms, which may include for example name-calling, aspersion, vulgarity, or use of pejoratives^1^. However, there is no objective definition of what is or is not uncivil communication. Communication that would be considered uncivil in one environment (e.g., on a news site’s comment section) might be seen as perfectly appropriate in another (e.g., in a private chat group). For example, uncivil discourse has been found to be equally common on Facebook and Twitter, but intolerant discussions occur less often on Facebook than on Twitter, where people communicate mainly with strangers^16^. Furthermore, what one person considers uncivil may be seen as civil by someone else^1^. Incivility is thus necessarily a subjective term, shaped not only by group norms, but also by individuals’ subjective evaluation of those norms.

In the current study, we use a similar definition of incivility as Frischlich et al.^6^. They define incivility as a communication that a person subjectively judges as having an unnecessarily disrespectful or offensive tone. The incivility can be aimed at other people, or towards specific topics and the content can be in any format, such as text, image, or video. With uncivil communication, we refer both to producing and spreading uncivil content^6^. Uncivil communication is set apart from a related term cyberbullying^17^ in that it is not necessarily intended to harm, and it is not necessarily a repeated behavior.

#### Online disinhibition

One of the reasons why uncivil online communication is so common is that online environments tend to lead to reduced behavioral constraints^10,18^. Simply put, people may do things online that they would not do in face-to-face interaction. This phenomenon has been named as the “online disinhibition effect”^10^. In the original description of the phenomenon, Suler^10^proposed that the online disinhibition effect leads to two, seemingly opposite behavioral outcomes. “Benign disinhibition” refers to seemingly positive outcomes, such as an increased tendency to share personal thoughts, or to help strangers in online settings. “Toxic disinhibition” refers to negative behavioral outcomes, such as an increased tendency to use rude language or to express hatred. These opposite outcomes, benign and toxic disinhibition are seen as two sides of the same coin, reflecting the same underlying behavioral disinhibition process^10,19^. Suler^10^proposed that online disinhibition is a result of several characteristics of online interactions that separate them from face-to-face communication (e.g., asynchronicity, relative anonymity, invisibility of the interaction partners). Later empirical research has confirmed that many of these characteristics indeed increase both uncivil behaviors (e.g^18,20,21^). , and experiences of online disinhibition^22^.

Importantly, features of the used online media are not the only factors determining the level of disinhibition, but disinhibition also varies substantially across individuals^10,19^. In other words, online environments lead to more behavioral disinhibition in some people than others. Individuals who report high levels of online disinhibition have an increased likelihood of expressing hate online^23,24^, and of engaging in cyberbullying^25^, trolling^19^, and online sexual harassment^26^. Experienced online disinhibition is typically measured with one of several questionnaires developed for this purpose (e.g^19,25^). , , as we also do in the current study.

#### Personality, empathy, and uncivil communication

Identifying factors that predict online disinhibition and uncivil behavior is crucial for understanding online incivility. There is ample evidence that individual’s personality and level of empathy play a role in influencing online behavior. Many of the studies that have examined the relationship between personality and online incivility have focused on the Big Five and Dark Triad models. It has been found that “dark personality traits” (i.e., psychopathy, narcissism, machiavellianism) correlate with uncivil online communication, aggressive online behavior, trolling, and experienced online disinhibition^5–7,27^. However, in the study of Koban et al.^28^ none of the Dark Triad Traits were statistically significantly related to the intention to comment uncivilly.

The five-factor model of personality^29^is one of the most comprehensive personality theories. In this model personality is described with five different dimensions: openness, conscientiousness, extraversion, agreeableness, and neuroticism. Prior research has shown that conscientiousness and agreeableness are negatively related, and extraversion is positively related to cyberbullying^30,31^. Additionally, Koban et al.^28^ found that openness and agreeableness negatively predicted uncivil commenting intentions.

Cognitive empathy refers to the ability to comprehend others’ affective states^32–34^. Affective empathy, on the other hand, means the capability to vicariously experience others’ affective states^32,34,35^. In the current study, we measured both of these core components: cognitive and affective empathy. It has been found that low cognitive empathy is associated with experienced online disinhibition in adolescents^5^. Additionally, Wright et al.^23^ found that callous-unemotional personality traits (characterized by lack of empathy, remorse, and guilt) predicted cyberbullying in adolescents.

#### Emotions, emotion regulation, and uncivil communication

While earlier research partially explains inter-individual variation in uncivil online behavior, much of this variation remains to be explained. We suggest that uncivil communication could further be explained by individual differences in emotion regulation processes. It is widely accepted that emotions are key motivators of intra- and inter-individual behavior, both in face-to-face interaction and on the internet^36,37^. In online discussions, emotional messages influence the direction the conversation will take. Emotionally toned messages generally cause emotional reactions in the readers^38,39^and may result in both further emotional content exchanges^8,40^and drift the discussion off-topic^41^. Further, if reading messages evokes intense negative emotions, people are more likely to speak out rather than remain silent regardless of the topic of the discussion or of how hostile or friendly the discussion climate is^42^. Experimental studies have also demonstrated a causal link between negative emotions and uncivil communication. Cheng et al.^9^ found that triggering negative emotions with experimental manipulations increased trolling when compared to triggering of positive emotions. Correspondingly, Seering et al.^43^ reported that user interface elements that evoke positive emotions as compared to no user interface intervention, increased the positivity of written messages. Taken together, these studies suggest that the more negative emotions people experience, the more likely they are to engage in uncivil communication.

Importantly, people are not passively at the mercy of their emotional reactions, but instead, they constantly regulate their emotions. Emotion regulation refers to the various psychological processes that modulate the quality, intensity, or duration of emotions^44–46^. This includes both explicit, effortful attempts at altering emotions, as well as implicit, relatively automatic emotion regulation processes^46^. Emotion regulation is important in practically all domains of life. Use of adaptive emotion regulation strategies, such as acceptance and cognitive reappraisal, predicts, for instance, better general well-being^47^, mental health^48^, and satisfaction of relationship^49^.

In one of the few papers that have investigated how emotion regulation processes shape online interactions, Fan et al.^50^investigated how the implicit emotion regulation process known as affect labeling (naming emotions) affects the emotions of Twitter users. They found that after users had named their emotional state, the tone of their subsequent posts changed towards a more neutral state than before naming of the emotion. This suggests that successful emotion regulation during social media use can lead users to act in a calmer manner. It could thus be expected that people with deficiencies in emotion regulation skills would be particularly prone to act uncivilly. Consistent with this, it was found that adolescents with emotion regulation difficulties are more likely than others to engage in cyberbullying^51,52^. However, to our knowledge, no studies have investigated associations between emotion regulation, online disinhibition, and uncivil communication.

The present study examined whether individual differences in emotion regulation beyond variation explained by personality and empathy would predict uncivil communication. We measured emotion regulation using two approaches. First and foremost, we measured difficulties in emotion regulation^53,54^. According to Gratz and Roemer^54^, difficulties in emotion regulation may occur in six domains: (1) nonacceptance of emotions, (2) difficulties in engaging in goal-directed behaviors when distressed, (3) difficulties in controlling impulses when distressed, (4) lack of awareness of emotions, (5) limited access to effective emotion regulation strategies, and (6) lack of clarity of emotions. In addition to emotion regulation difficulties, we also measured the habitual use of two different and most well-researched emotion regulation strategies: cognitive reappraisal and expressive suppression^45,47^. Reappraisal involves cognitive processing of emotion-evoking situations to alter their influence on emotions (e.g., interpreting that a hostile message was written by someone who was just having a bad day). Reappraisal is typically considered an adaptive emotion regulation strategy. Suppression, on the other hand, is characterized by concealment of emotional expressions (e.g., keeping a still facial expression when experiencing anger). Suppression is usually considered a relatively maladaptive strategy. There is growing evidence that reappraisal reliably downregulates negative emotional experiences, but suppression is not as effective^55^.

## Methods

### Overview of the study

We conducted an online survey to examine associations between emotion regulation, online disinhibition, and uncivil communication. Respondents reported whether they had engaged in uncivil communication during the past three months, and filled in scales measuring personality, empathy, experienced online disinhibition, difficulties in emotion regulation, and use of different emotion regulation strategies (reappraisal and suppression). As disinhibition is associated with incivility, we anticipated that online disinhibition could be a possible mediating mechanism by which emotion regulation would predict uncivil communication. Therefore, we subjected the data to a mediation analysis. We were particularly interested in whether emotion regulation would explain uncivil communication, beyond what is explained by personality and empathy. Therefore, the effects of personality and empathy were controlled for in the analysis.

### Statement on research ethics

At the time of conducting this research, anonymous surveys did not require formal review by a research ethics committee under Finnish research governance, in which the Declaration of Helsinki defines applicability to research on identifiable human data. The survey followed ethical research practices as defined, for example, by the Finnish National Board on Research Integrity and Finnish law (i.e., voluntary participation; reassurance of anonymity, data protection, and confidentiality; advance information on purpose and content; provision of contact details of the research team; and full disclosure of involved organizations). This information was summarized on the first page of the online survey. Anonymous electronic consent to voluntary participate was required to begin the survey, but no signatures were obtained.

### Sample

The sample size was determined based on a power analysis, conducted using the pwr package for R^56^. We calculated a sample size sufficient for detecting a typical correlation in an individual difference study (*r*= .19^57^;). We anticipated this to be a reasonably conservative estimate, as earlier studies on similar topics have reported larger correlations (e.g., *r* = .3 between emotion regulation difficulties and cyberbullying in Jiang et al.^52^). The power analysis suggested a sample size of *N*= 215 at α = 0.05 and power = 0.80^58^. This number of respondents were recruited via the participant recruitment platform Prolific^59^. The inclusion criteria were English as first language and a minimum of 90% approval rate on Prolific. Respondents received £1.88 as compensation for their participation. Eleven respondents failed at least in one of the three attention check items included in the survey^60^ and were thus removed from all analyses and replaced with new respondents. The median response time in the analyzed sample was 10 min and 42 s. The characteristics of the analyzed sample are presented in Table 1.

**Table 1:** Characteristics of the sample.

	M	SD
Age	33.2	11.6
Gender	*n*	%
Male	78	36.3
Female	133	61.9
Other	4	1.9
Education	*n*	%
Not applicable	2	0.9
Secondary	19	8.8
High school	49	22.8
Technical / community college	23	10.7
Undergraduate	93	43.3
Graduate	26	12.1
Doctorate	3	1.4
Frequency of posting content	*n*	%
Never	61	28.4
Less than once a month	52	24.2
At least once a month	51	23.7
At least once a week	39	18.1
At least once a day	12	5.6

### Survey

The respondents filled in a survey that was described as investigating “online and offline behavior”. The survey was conducted with the LimeSurvey web application. It consisted of scales measuring uncivil communication and various individual traits (see below). The survey also contained a few additional questions about whether they had read or posted user-generated content in the past three months, what types of platforms they had used, and whether they usually post to anonymous services or services that display users’ real names or nicknames. Only the scales on uncivil communication and individual traits are reported in the current study.

#### Outcome variable - uncivil communication

Uncivil communication was measured using the scale developed by Frischlich et al.^6^. In the scale, respondents evaluate the frequency of various kinds of responses to uncivil content, such as posting, “liking”, reporting, and ignoring such content during the past three months. The scale consisted of ten items, all on a 5-point scale (ranging from “Never” to “At least once per day”). We analyzed responses identically to Frischlich et al.^6^: In the analysis, we only included the four items representing uncivil communication, that is, how often the participants had posted, shared, showed, and liked uncivil content (Cronbach’s alpha = 0.672). While Frischlich et al.^6^ called this “uncivil participation,” we think that the term “uncivil communication” would be more appropriate as posting, sharing, and showing are generally active forms of communication, and liking, while leaning toward participation, can be a subtle form of communication. Just like in the earlier study, the distribution of the means of the items representing uncivil communication were highly positively skewed. Thus, just like Frischlich et al.^6^, we dummy-coded whether participants had engaged in these types of behaviors (0 = no, 1 = yes). In the current sample, 48.4% of respondents reported having engaged in uncivil communication in the past three months. In turn, we can see from Table 1 that 47.4% have posted at least once a month while 52.6% of the respondents have rarely (less than once a month) or never posted a comment online. Table 2 illustrates the connection between the frequency of posting and engaging in uncivil communication during the past three months. It shows that respondents having engaged in uncivil communication are not only people who post more often, but also many of infrequent commenters had engaged in uncivil communication.

**Table 2:** Distribution of the respondents (%) regarding frequency of posting and engaging uncivil communication during past three months.

		Uncivil communication	
		No	Yes
Frequency of posting	Never	41	20
	Less than once a month	25	27
	At least once a month	24	27
	At least once a week	18	21
	At least once a day	3	9

#### Mediating variable - online disinhibition

Online disinhibition was measured using the Measure of Online Disinhibition^19^. It consists of 12 items, including statements such as “I am more assertive online than I am offline” and “My behaviors online are less restricted than in person”, all evaluated on a 5-point Likert scale. All items load on the same factor (Cronbach’s alpha = 0.921). For the analysis, we averaged the items and then mean-centered the resulting online disinhibition score.

#### Predictor variables - emotion regulation

Difficulties in emotion regulation were measured using the brief version of the Difficulties in Emotion Regulation Scale (DERS-16)^53^. It consists of sixteen items (e.g., “I have difficulty making sense out of my feelings”, “When I’m upset, I have difficulty getting work done”), measured on a 5-point Likert scale. For the analysis, we averaged the sixteen items to create an index of difficulties in emotion regulation (Cronbach’s alpha = 0.945). The score was then mean-centered.

To measure the habitual use of different emotion regulation strategies, we used the Emotion Regulation Questionnaire (ERQ)^47^. The questionnaire consists of ten items measuring the use of cognitive reappraisal (Cronbach’s alpha = 0.881) and expressive suppression (Cronbach’s alpha = 0.723). The items representing the two factors were averaged to create the respective indices, and then mean-centered.

#### Covariates - personality and empathy

Personality traits were measured using the Big Five Inventory-10^61^. It is a very short, 10-item questionnaire measuring personality traits according to the five-factor model of personality. The short scale was chosen to keep the survey brief. All items are on a 5-point scale. Items were reverse scored where necessary and averaged to create indices of each of the five factors. The scores were mean centered for the analysis. Reliability of the factors were examined using the Spearman-Brown coefficient, as it is considered the best statistic for assessing the reliability of two-item scales^62^. Coefficients for the agreeableness and openness subscales were unacceptably low (0.384 and 0.350, respectively), and were thus excluded from the analysis. Coefficients for the other subscales were 0.612 for the extraversion, 0.523 for the conscientiousness, and 0.579 for the neuroticism. These coefficients are also quite low, but can be considered acceptable/sufficient^63^.

Empathy was measured with the Basic Empathy Scale in Adults^32^. The questionnaire consists of 20 items, measured on a 5-point scale. The items were reverse scored where necessary, and then averaged to create indices of affective empathy (Cronbach’s alpha = 0.866) and cognitive empathy (Cronbach’s alpha = 0.850). The scores were mean centered for the analysis.

### Data analysis

We conducted a mediation analysis with a regression-based approach, using the PROCESS macro for SPSS (version 4.0)^64^. In this analysis, a mediation model is constructed by conducting a series of regression analyses to estimate associations between predictor variables, mediators, and outcome variables. Ordinary least squares and logistic regression models are used for continuous and dichotomous consequent variables, respectively. Covariates can be included in the mediation model to control their effects. Indirect effects are calculated by taking the product of the (predictor variable → mediator) and (mediator → outcome variable) regression coefficients. Confidence intervals (CI) for the indirect effects are estimated with bootstrapping.

We constructed a single mediation model with three predictor variables (emotion regulation difficulties, reappraisal, suppression), one mediator (online disinhibition), one outcome variable (uncivil communication, dummy-coded variable) and five covariates (conscientiousness, extraversion, neuroticism, cognitive empathy, affective empathy). We examined 95% CIs for the indirect effects based on 100,000 bootstrap samples.

## Results

Tables 3 and 4 report the means and standard deviations of each studied variable and single correlation coefficients between variables as basic statistics.

**Table 3:** Mean and standard deviation of the studied variables.

	Mean	Std. Deviation
Extraversion	2,93	0,95
Agreeableness	3,46	0,83
Conscientiousness	3,60	0,85
Neuroticism	3,22	1,01
Openness	3,57	0,88
Disinhibition	2,27	0,92
AffectiveEmpathy	3,51	0,68
CognitiveEmpathy	3,97	0,55
Reappraisal	4,71	1,03
Suppression	3,95	1,19
Emotion Regulation Difficulties	2,41	0,87
Uncivil (dummy)	0,48	--

**Table 4:** Correlations of the variables.

		Extraversion	Agreeableness	Conscientiousness	Neuroticism	Openness	Disinhibition	Affective Empathy	Cognitive Empathy	Reappraisal	Suppression	Emotion Regulation Difficulties
Agreeableness		−0,039	--									
	Sig.	0,569										
Conscientiousness		0,092	,267^**^	--								
	Sig.	0,181	0,000									
Neuroticism		-,303^**^	−0,065	-,316^**^	--							
	Sig.	0,000	0,340	0,000								
Openness		0,000	−0,097	−0,032	0,105	--						
	Sig.	0,996	0,156	0,639	0,124							
Disinhibition		-,249^**^	−0,088	-,295^**^	,326^**^	−0,022	--					
	Sig.	0,000	0,200	0,000	0,000	0,745						
Affective Empathy		−0,012	,177^**^	0,048	,241^**^	,247^**^	0,011	--				
	Sig.	0,864	0,009	0,487	0,000	0,000	0,873					
Cognitive Empathy		0,091	0,066	,210^**^	-,159^*^	,211^**^	−0,016	,445^**^	--			
	Sig.	0,184	0,337	0,002	0,020	0,002	0,818	0,000				
Reappraisal		0,095	0,105	,182^**^	-,245^**^	0,074	−0,066	,168^*^	,363^**^	--		
	Sig.	0,164	0,125	0,008	0,000	0,283	0,338	0,014	0,000			
Suppression		-,232^**^	−0,063	-,139^*^	0,072	−0,076	,225^**^	-,168^*^	-,144^*^	0,059	--	
	Sig.	0,001	0,359	0,042	0,291	0,264	0,001	0,013	0,035	0,386		
Emotion Regulation Difficulties		−0,133	−0,111	-,364^**^	,584^**^	0,048	,367^**^	,146^*^	-,188^**^	-,283^**^	,279^**^	--
	Sig.	0,052	0,104	0,000	0,000	0,485	0,000	0,032	0,006	0,000	0,000	
Uncivil (dummy)		0,007	−0,069	−0,102	0,037	−0,082	,190^**^	−0,008	0,026	-,154^*^	0,085	0,127
	Sig.	0,916	0,315	0,134	0,588	0,230	0,005	0,911	0,700	0,024	0,214	0,062

**Fig. 1:**
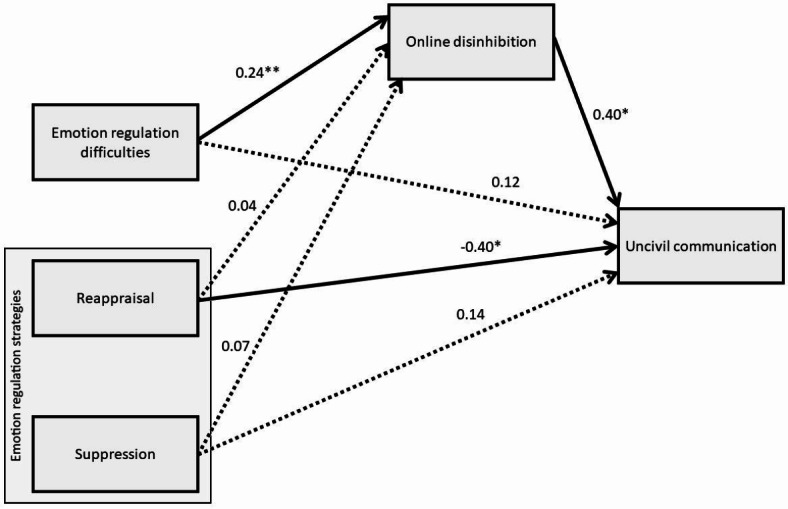
The resulted mediation model. The solid and dashed lines indicate significant and non-significant associations, respectively. The numbers represent regression coefficients. Where uncivil communication is the consequent variable, the coefficients are in log-odds metric. Covariates are omitted from the figure for the sake of clarity. **p < .01, *p < .05.

The associations between the emotion regulation variables (predictors), online disinhibition (the mediator), and uncivil communication (the outcome variable) are depicted in Fig. 1. Estimates of the indirect effects are shown in Table 5. For the regression model with disinhibition as the outcome variable, the model summary is F 7.9201, R 0.4850, R2 0.2352, MSE 0.6699, df1 8.0, df2 206.0, p 0.0000. For the model with incivility as the outcome variable, the model summary is −2LL 280.1480, Model LL 17.6773, df 9.0, p 0.0391, McFadden 0.0594, CoxSnell 0.0789, Nagelkrk 0.1053. Associations between the covariates (personality and empathy variables) and online disinhibition and uncivil communication are shown in Table 6.

**Table 5:** Bootstrap estimates of indirect effects of emotion regulation variables on uncivil communication via online disinhibition. The coefficients are in log-odds metric.

	Effect	SE	95% CI
Emotion regulation difficulties	0.098	0.069	[0.001, 0.267]
Reappraisal	0.014	0.030	[−0.036, 0.086]
Suppression	0.029	0.030	[−0.016, 0.101]

**Table 6:** Coefficients for the associations between personality and empathy variables and online disinhibition and uncivil communication. Where uncivil communication is the consequent variable, the coefficients and confidence intervals are in log-odds metric.

Online disinhibition					
	Coefficient	SE	*t*	*p*	95% CI
Extraversion	−0.15	0.06	−2.36	0.019	[−0.278, −0.025]
Conscientiousness	−0.19	0.07	−2.57	0.011	[−0.327, −0.043]
Neuroticism	0.13	0.08	1.72	0.087	[−0.019, 0.278]
Affective empathy	−0.14	0.10	−1.43	0.154	[−0.342, 0.054]
Cognitive empathy	0.25	0.13	1.97	0.051	[−0.001, 0.494]
Uncivil communication					
	Coefficient	SE	*Z*	*p*	95% CI
Extraversion	0.14	0.166	0.86	0.391	[−0.183, 0.467]
Conscientiousness	−0.11	0.188	−0.57	0.572	[−0.474, 0.262]
Neuroticism	−0.16	0.194	−0.54	0.399	[−0.545, 0.217]
Affective empathy	0.00	0.259	0.00	> 0.999	[−0.508, 0.508]
Cognitive empathy	0.43	0.327	1.32	0.186	[−0.208, 1.073]

When the scales with low internal consistency, agreeableness and openness, are included in the model, the indirect effect of emotion regulation difficulties on uncivil communication via online disinhibition is slightly smaller, and notably, the confidence interval includes zero (effect = 0.096, SE = 0.07, 95% CI [−0.00, 0.27]).

Most importantly, the model showed that emotion regulation difficulties predicted higher levels of online disinhibition, which, in turn predicted uncivil communication. The 95% CI for this indirect effect was entirely above zero. Additionally, the results showed that the use of reappraisal predicted lower levels of uncivil communication directly. No other significant associations involving emotion regulation variables were found. As for the covariates, it was found that extraversion and conscientiousness predicted lower levels of online disinhibition. No other significant associations were found.

## Discussion

Our analysis implies a clear statistical association: the more the respondents reported emotion regulation difficulties, the more they experienced online disinhibition, that is, weakened online behavioral restraints. Higher online disinhibition then predicted a higher likelihood of reporting uncivil online communication. This result expands on earlier knowledge on the role of user emotions in shaping online behavior. Prior research suggests that encountering emotion evoking content can lead people to post emotional messages^8^, to troll other users^9^, and to shift the discussion off-topic^41^. It is likely that emotion regulation has a central role in this process: individuals who successfully manage to regulate their emotions are better in inhibiting their behavior than those not so successful in regulation. Better regulation in turn associates to calm and civil communication (see also^50^). In contrast, individuals who have difficulties with emotion regulation are at an increased risk to lash out, resulting in uncivil communication.

Importantly, the results pertaining to emotion regulation strategies showed that especially higher use of reappraisal predicted lower levels of uncivil communication directly. Suppression as a strategy was not statistically significantly associated to uncivil communication. Reappraisal is a cognitive-linguistic strategy in which people change their emotions by reformulating the meaning of an emotion-eliciting event^65^. It is considered an adaptive emotion regulation strategy because its frequent use is associated with enhanced control of emotion, interpersonal functioning, and well-being^47^. The results support the previous findings that reappraisal is an effective strategy in downregulating negative emotional experiences^55^, and suggest that reappraisal skills have important role in regulating emotions and affecting interpersonal communication also in online environments.

Importantly, the observed associations between emotion regulation and uncivil communication were significant even when controlling for personality and empathy. Previous research has demonstrated that dark personality traits and low empathy are associated with uncivil online behavior^5–7^. Further, previous research has found that some of the Big five personality traits are related to cyberbullying^30,31^or uncivil communication intentions^28^. The current result suggests that emotion regulation processes predict inter-individual variation in uncivil communication, beyond the variation in personality and empathy.

The personality and empathy measurements also produced noteworthy findings. High conscientiousness and extraversion predicted low levels of online disinhibition. This consolidates prior research, which has found that dark personality traits such as psychopathy correlate with online disinhibition^5,7^. Dark personality traits and traits of the five-factor model are distinct, but partially overlapping concepts. For instance, conscientiousness negatively correlates with dark personality traits^66^, and therefore the current results are in line with earlier research. In the present study neither cognitive nor affective empathy predicted online disinhibition or uncivil communication. This contrasts with one earlier study, which reported that cognitive empathy negatively correlated with online disinhibition in adolescents^5^. One possible explanation as to why the result was not replicated is the different samples (adolescents vs. adults).

### Limitations, future work, and practical implications

A limitation of the study was that two of the five personality scales, agreeableness and openness, had unacceptably low internal consistency in our data, and were thus excluded from the analyses. Their exclusion is noteworthy, as openness and agreeableness may have impact on online behavior. However, we believe the exclusion of these scales was justified. While we used a personality scale which has demonstrated sufficient psychometric properties in earlier research^61^, the low internal consistency suggests the two scales did not satisfactorily measure the underlying personality dimensions in our study.

Additionally, while the three retained subscales—Extraversion, Neuroticism, and Conscientiousness—were included in the mediation model, their internal consistency was also quite low. It has been noted that these subscales are best represented by their 2-item versions^61^, which influenced our decision to include them. However, the low internal consistency of these scales should be taken into account when interpreting the results and understanding the connection between personality traits and uncivil communication. Future studies should consider using the original Big Five Inventory, consisting of 44 items, as this longer version has demonstrated higher reliability^61^.

Another limitation of our study is our use of Prolific to recruit responders. Prolific has limitations such as sample bias, where participants tend to be younger and more educated, limiting generalizability. Also, the response quality may have been affected by survey fatigue or speeding up in completing the survey. In hindsight, we believe it was good that we also included individuals who never posted online comments or posted rarely in the sample. While this may be considered as a limitation, we argue that online behavior is more than just posting comments. In the present study, almost half of the infrequent commenters reported having engaged in uncivil communication in the past three months. Instead of posting they have likely shared, showed, or liked other people’s uncivil content and thereby engaged in uncivil behavior. Nevertheless, future studies could focus on only individuals who are frequent posters to dwell deeper on the question of posting frequency and emotion regulation.

Based on the current study, we cannot draw firm conclusions about causal relationships between the success of emotion regulation and online behavior. However, experimental studies have established a causal link between users’ emotions and uncivil commenting behavior^8,9^. Thus, it seems likely that much of the uncivil communication online can result from a failure to regulate emotions. To determine that the associations between emotion regulation, online disinhibition and uncivil communication are causal in nature, experimental or longitudinal studies would be needed.

The findings of the present study not only broaden our understanding of who engages in uncivil online communication, but also suggest possible avenues for mitigating this type of behavior. We suggest development of interventions for promoting users’ emotion regulation, thereby potentially mitigating uncivil communication. Similarly, Kiskola et al.^67^recently proposed user interface designs that might help users recognize and regulate their emotions. Future user interface designs could make use of the current finding that especially reappraisal predicted low likelihood of uncivil communication. For example, when encountering emotion evoking content on social media, the user could be nudged to reappraise the intentions of the person who had posted the content, possibly reducing the inclination to respond uncivilly. Further, as the present study mostly focused on forms of incivility that relate to speech-based norms, and only indirectly addressed violations of the inclusion-based norms^11^, future studies could focus more on the inclusion based norms.

### Conclusion

The current study showed that individuals with emotion regulation difficulties are particularly prone to engage in uncivil communication online. It seems likely that a significant portion of uncivil communication results from users’ failure to regulate their emotions. Thus, to mitigate some of the negative outcomes of social media, it would be important to develop methods to support emotion regulation while engaging in online interactions on social media.

## Data Availability

The data that support the findings of this study are openly available in Fairdata.fi at https://doi.org/10.23729/de438b0d-072f-4f2e-a8b5-204576ab76ef.
